# Various Nodal Lines in P6_3_/mmc-type TiTe Topological Metal and its (001) Surface State

**DOI:** 10.3389/fchem.2021.755350

**Published:** 2021-09-28

**Authors:** Peng Lin, Fang Fang, Li Zhang, Yang Li, Kai Wang

**Affiliations:** ^1^ Engineering and Technology Center, The Fourth Medical College of Harbin Medical University, Harbin, China; ^2^ Changchun Institute of Technology, Changchun, China; ^3^ Nanoscience and Engineering and Technology Electrophysiology Research Center, The Fourth Medical College of Harbin Medical University, Harbin, China

**Keywords:** DFT study, tite, nodal line states, surface states, SOC

## Abstract

Searching for existing topological materials is a hot topic in quantum and computational chemistry. This study uncovers P6_3_/mmc type TiTe compound—an existing material—is a newly discovered topological metal that hosts the various type of nodal line states. Different nodal line states normally exhibit different properties; they may have their individual applications. We report that TiTe hosts I, II, and hybrid type nodal line (NL) states at its ground state without chemical doping and strain engineering effects. Specifically, two type I NLs, two hybrid-type NLs, and one Γ—centered type II NL can be found in the k_z_ = 0 plane. Moreover, the spin-orbit coupling induced gaps for these NLs are very small and within acceptable limits. The surface states of the TiTe (001) plane were determined to provide strong evidence for the appearance of the three types of NLs in TiTe. We also provide a reference for the data of the dynamic and mechanical properties of TiTe. We expect that the proposed NL states in TiTe can be obtained in future experiments.

## Introduction

Searching for topological materials in realistic materials in quantum and computational chemistry is a hot research topic. Topological materials (TMs) (Cava et al., 2013; Kong and Cui, 2011; Xu et al., 2015; Strambini et al., 2016; Wang et al., 2017; Banik et al., 2018; Kageyama et al., 2018; Schoop et al., 2018; Culcer et al., 2020; Kumar et al., 2020; Li and Xia, 2020; Xu et al., 2020) enjoy nontrivial band-crossings (BCs) in their low-energy region, giving rise to novel fermionic excitations. A series of TMs, including nodal-point (Alcón et al., 2017; Fu et al., 2018a; Kong et al., 2018; Jin et al., 2019a; Jin et al., 2019b; Wang et al., 2019; Fang et al., 2020; Zhang et al., 2020), nodal-line (Chen et al., 2018; Zhou et al., 2018; Li et al., 2019; Liu et al., 2019; Sankar et al., 2019; Tang et al., 2019; Xu et al., 2019; Yi et al., 2019; Wang et al., 2020a; Zhao et al., 2020), and nodal-surface (Wu et al., 2018; Qie et al., 2019; Wang et al., 2020b) materials, have been predicted *via* symmetry and first-principle analysis. Some of them have been verified *via* experiment.

Recently, many chemists and physicists have focused on studying the nodal line (NL) type materials. The NL-type materials are very important because they can enjoy more sub-types than other types of topological materials; moreover, different sub-types generally have their physical behaviors. Many NL materials with different NL shapes have been proposed, including nodal ring (Zhang et al., 2018a), nodal chain (Bzdušek et al., 2016), nodal link (Yan et al., 2017), nodal knot (Bi et al., 2017; Ezawa, 2017), and nodal net materials (Wang et al., 2018a; Fu et al., 2018b; Feng et al., 2018). Different shapes of the NLs usually exhibit different electronic and optical behaviors. Moreover, NLs can normally be classified into the I, II, and hybrid types (Jin et al., 2020) according to the slope of the bands around the band-crossing points (BCPs).

**FIGURE 1:**
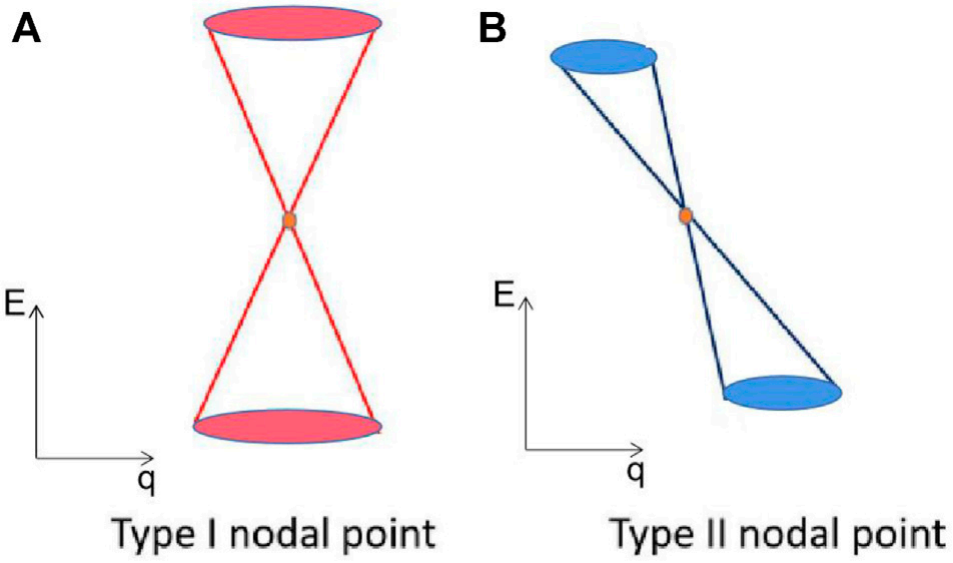
Illustration of the type **(A)** I and **(B)** II NLs.

The I type NL is composed of all the type I BCPs, and the II type NL is formed by the type II BCPs. However, the hybrid-type NL contains I and II type NLs simultaneously. The illustration of I and II type BCPs are shown in Figures 1A,B, respectively. The I type BCPs show a traditional conical dispersion, whereas the II type BCPs show a titled dispersion.

A series of materials (Wang et al., 2018b; Wang et al., 2020c; Jin et al., 2020), type I or II NLs, have been studied *via* symmetry analysis and first-principles calculations. However, materials with I and II type NLs have rarely been explored in the literature. It is fascinating to investigate if I, II, and hybrid NLs can coexist in one material without strain, chemical doping, or other controlling methods.

**FIGURE 2:**
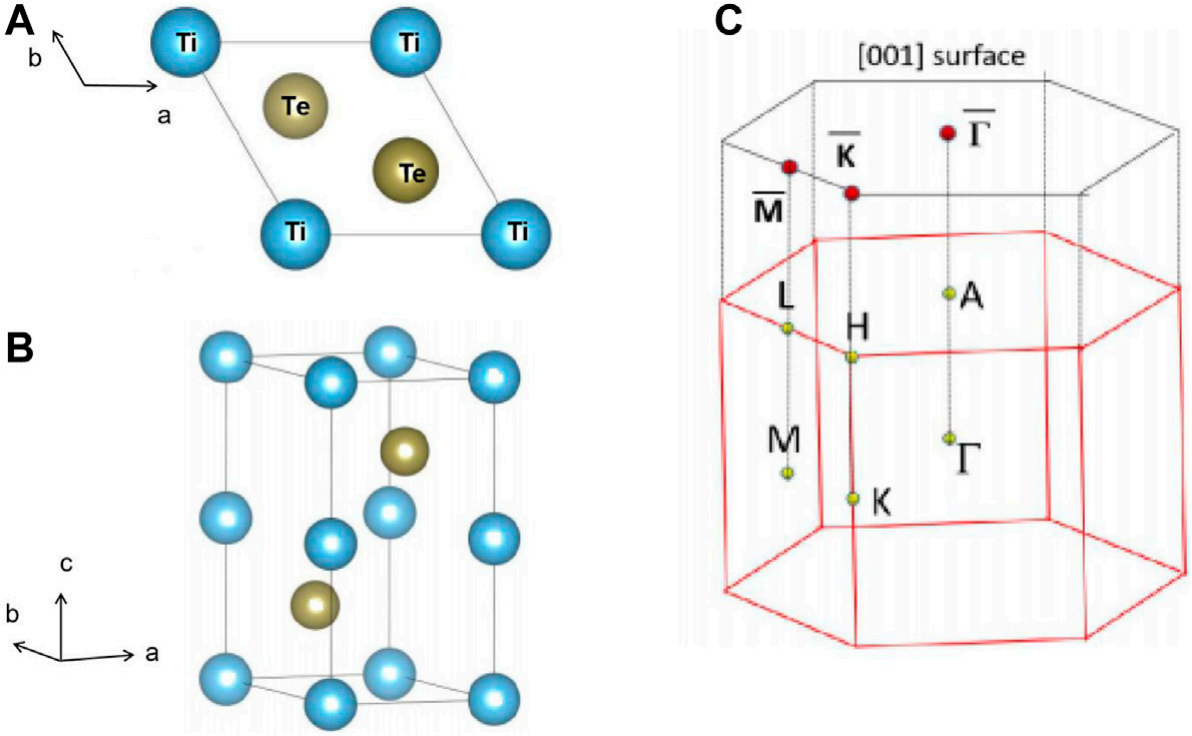
**(A,B)** Structural models. **(C)** The bulk and the (001) surface Brillouin zone.

In this work, we select P6_3_/mmc type TiTe material as an example and to show that the I, II, and hybrid types of NLs can coexist in realistic TiTe material (Ehrlish, 1949). The TiTe has already been realized in the experiment. We show that I, II, and hybrid NLs can be found in the kz = 0 plane of TiTe. The structural model of hexagonal P6_3_/mmc type TiTe with a primitive cell is exhibited in Figures 2A,B under different view sides. TiTe contains two Ti and two Te atoms, located at the (0 0 0)/(0, 0, 0.5) sites and the (1/3, 2/3, 0.25)/(2/3, 1/3, 0.75) sites, respectively. We optimized the lattice constants and the atomic positions based on the first-principle calculation. The obtained lattice constants of TiTe were a = b = 3.66 Å and c = 7.27 Å, agreeing well with the calculated values in the database^1^.

This study reports the band structures, phonon dispersions, and topological signatures of TiTe. We uncover that TiTe is an NL metal with one pair of type I NLs, one type II NL, and one pair of hybrid NLs in the kz = 0 plane. We also examine the influence of spin-orbit coupling (SOC) on the band structures. Finally, we calculate the projected spectrum on the (001) surface of TiTe show the occurrence of drum-head-like surface states connected to the BCPs. More details about the computational methods can be found in Supplementary Material.

## Dynamical Stability and Mechanical Stability

**FIGURE 3:**
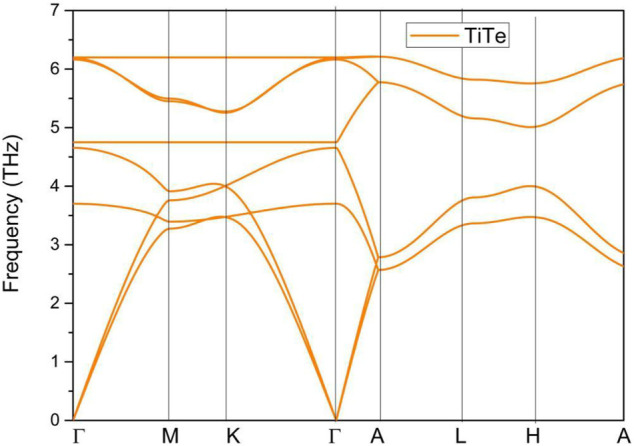
Phonon dispersion of TiTe bulk.

In this section, we present the study of the stabilities of TiTe with respect to dynamical and mechanical properties. Based on the bulk Brillouin zone and the selected symmetry points in Figure 2C. The phonon dispersion of TiTe was calculated through the force-constants method; the result is given in Figure 3. We conclude that the TiTe is dynamically stable because its phonon dispersion does not include imaginary frequencies.

Subsequently, the mechanical stability of TiTe is examined according to elastic stability criteria. TiTe has a P6_3_/mmc structure with five elastic constants—C_11_, C_12_, C_13_, C_33_, C_44_, and C_66_. The computed values of C_11_, C_12_, C_13_, C_33_, C_44_, and C_66_ were 133.543, 47.021, 78.611, 173.304, 43.206, and 107.550 GPa, respectively. We conclude based on the obtained elastic constants that they meet the criteria for elastic stability, as mentioned below:

i) C_11_ > |C_12_|;
ii) 2 × $C_{13}^{2}$ < C_33_(C_11_ + C_12_);
iii) C_44_ > 0.

Hence, TiTe is mechanically stable theoretically.

## Electronic Structures and Topological Signatures of Bulk TiTe

**FIGURE 4:**
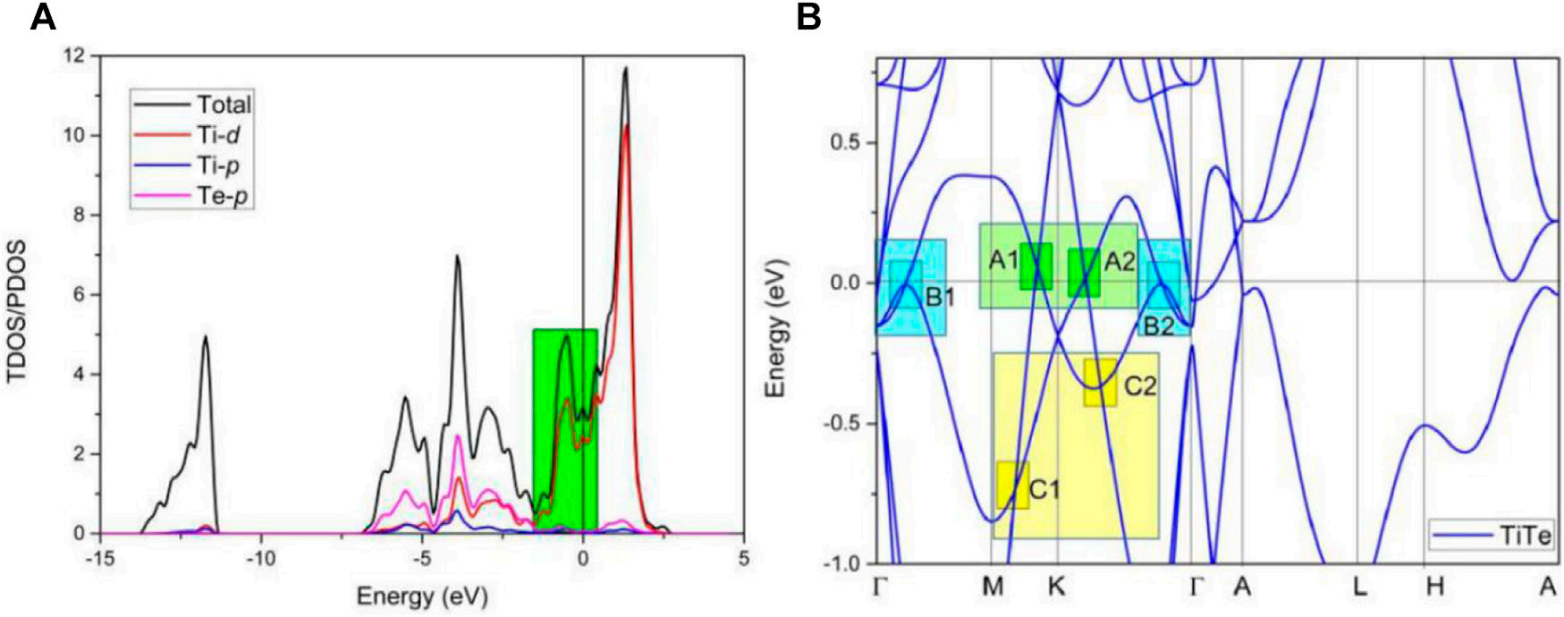
**(A)** Total and projected density of states and **(B)** band structure of TiTe bulk.

Figure 4A shows the calculated total and projected density of states (DOSs). We conclude that a small peak appears at the Fermi level (E_F_). Therefore, TiTe is a metallic material. The band structure of the TiTe metal is given in Figure 4B. We primarily focus on the bands closed to the E_F_. We observe that the Ti-d orbitals dominate the total DOSs in this region (-2 to 1 eV), as shown in Figure 4A with a green background. However, within the −5 to −2 eV energy range, the dominating factors contributing to the total DOSs are the Ti-d and Te-p orbitals. There exists a strong hybrid phenomenon between the Ti-d and Te-p orbitals in this energy range.

We show the band structure of the TiTe in Figure 4B ignoring the SOC. The band structure shows a series of BCPs above and below the E_F_. For clarity, we divided these BCPs into region A, region B, and region C, respectively. A, B, and C regions are marked by different colors. Two obvious BCPs—A1 and A2—located above the E_F_ can be found in region A. Two BCPs—B1 and B2—located very close to and below the E_F_ appear in region B. There are also two BCPs—C1 and C2—in region C. However, they are slightly further away from the E_F_ than the other BCPs—A1, A2, B1, and B2.

Different types of BCPs are discussed in regions A, B, and C. The two BCPs in region A are I type nodal points (NPs). Weng et al. (Weng et al., 2015) stated that these doubly degenerated crossing points (A1 and A2) are not isolated points; they should belong to a line. We conclude based on the plotted Brillouin zone of 3D bulk TiTe in Figure 2C that the A1 and A2 NPs are located in the kz = 0 plane.

**FIGURE 5:**
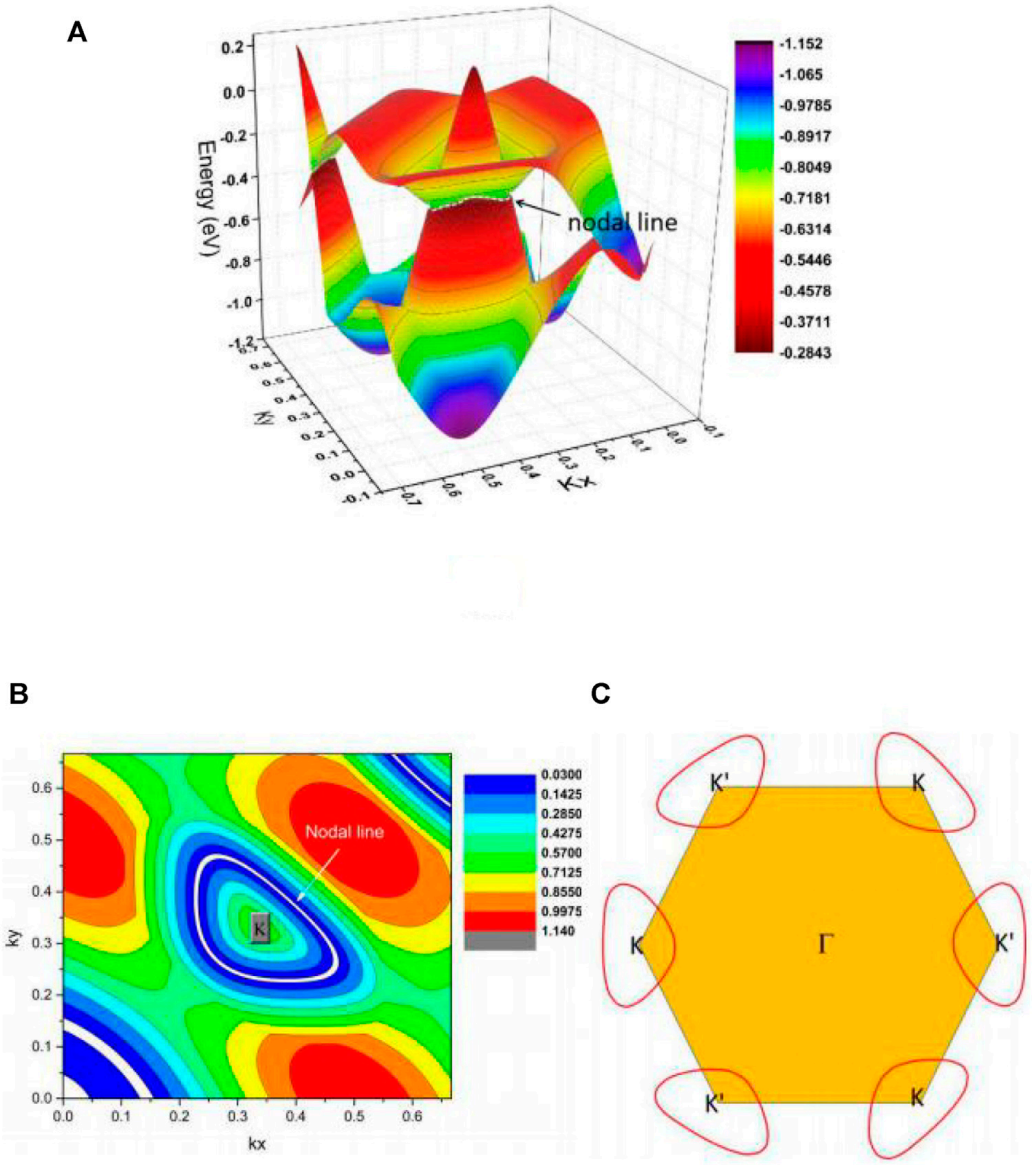
**(A)** 3D band dispersion in region A of the kz = 0 plane; **(B)** the shape of the NL in region A of the kz = 0 plane; and **(C)** illustration of one pair of type I NLs in the kz = 0 plane.

We show the K-centered 3D plotting of the band dispersion in region A of the kz = 0 plane in Figure 5A to demonstrate that the A1 and A2 NPs reside on an NL. We conclude that the energy variation of the NL in region A is very small. Such a flat NL is expected to host novel behaviors. Figures 5A,B show the highlighted NL (see the white dotted line) and the shape of the NL in region A, respectively. We conclude that the NL in region A is a type I. Furthermore, the NLs in region A are protected by two independent mechanisms: i) mirror symmetry and ii) inversion symmetry and time-reversal symmetry. TiTe possesses time-reversal symmetry; therefore, one more K′-centered NL should appear in the kz = 0 plane. The shape of one of the pairs of NLs, i.e., the K and K′ centered NLs, is shown in Figure 5C.

**FIGURE 6:**
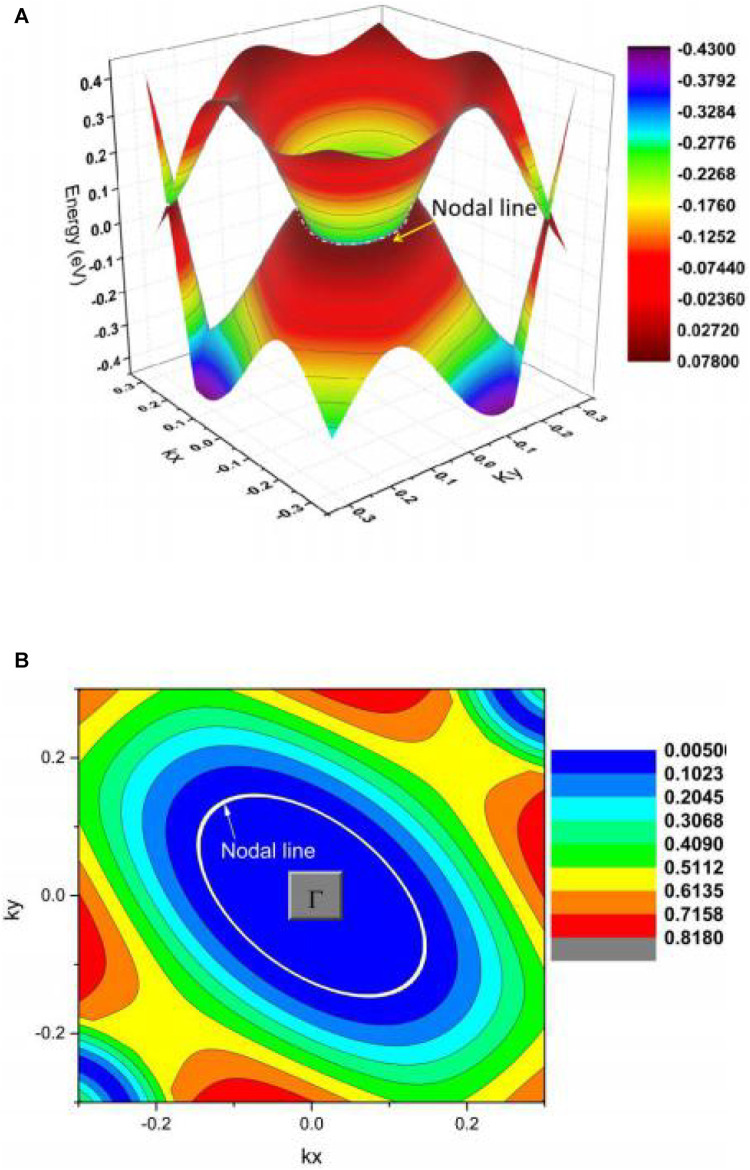
**(A)** Γ-centered 3D band dispersion in region B of the kz = 0 plane and **(B)** shape of the NL in region B of the kz = 0 plane.

In region B, two type II NPs, B1 and B2, also belong to a single NL and the Γ -centered 3D band dispersion in region B of the kz = 0 plane and the shape of the NL in region B are given in Figures 6A,B, respectively. We highlight NL by a white dotted line. This Γ -centered band dispersion has a small energy variation, similar to the NL in region A. Figure 6 shows that the NL in region B is type II.

**FIGURE 7:**
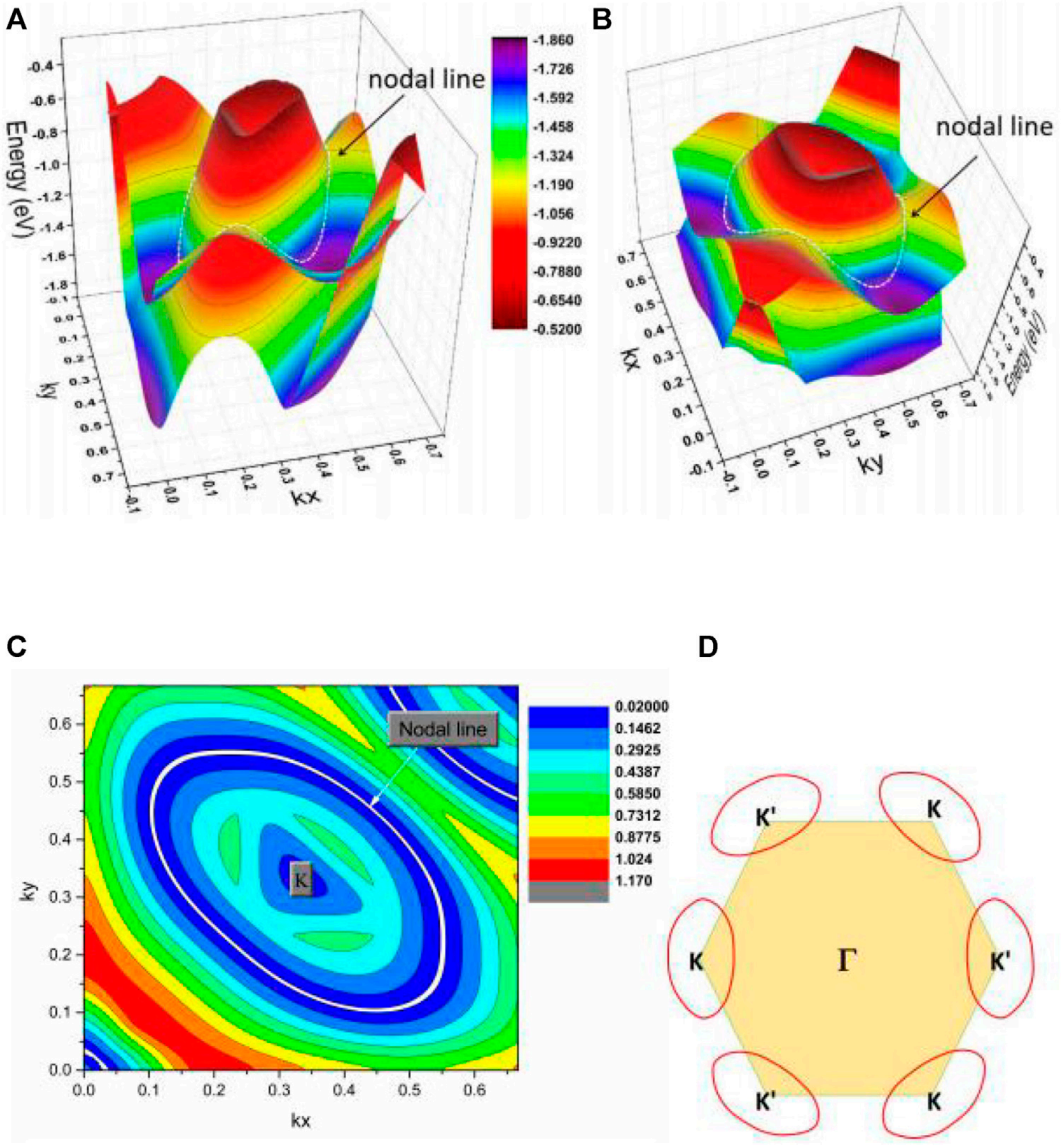
**(A,B)** K-centered 3D band dispersion in region C of the kz = 0 plane from different viewpoints. **(C)** The shape of the NL in region C of the kz = 0 plane. **(D)** Illustration of one pair of hybrid type NLs in the kz = 0 plane.

Finally, the K-centered 3D band dispersion in region C of the kz = 0 plane and the shape of the NL in region C are exhibited in Figures 7A–C to determine the topological signatures of the C1 and C2 NPs in region C. We conclude from the different viewpoints of the K-centered 3-D band dispersion that the energy variation of the NL is significantly large (from −0.8 to −0.3 eV). The reason for such a large energy variation is because it is a hybrid NL (Zhang et al., 2018b), containing type I and type II NPs at the same time. Figure 4C shows that BCP C1 is a type I; however, BCP C2 is type II. Moreover, another K′-centered hybrid NL should be located in the kz = 0 plane as required by the time-reversal symmetry (Figure 7D).

## Projected Spectrum on the TiTe (001) Surface

**FIGURE 8:**
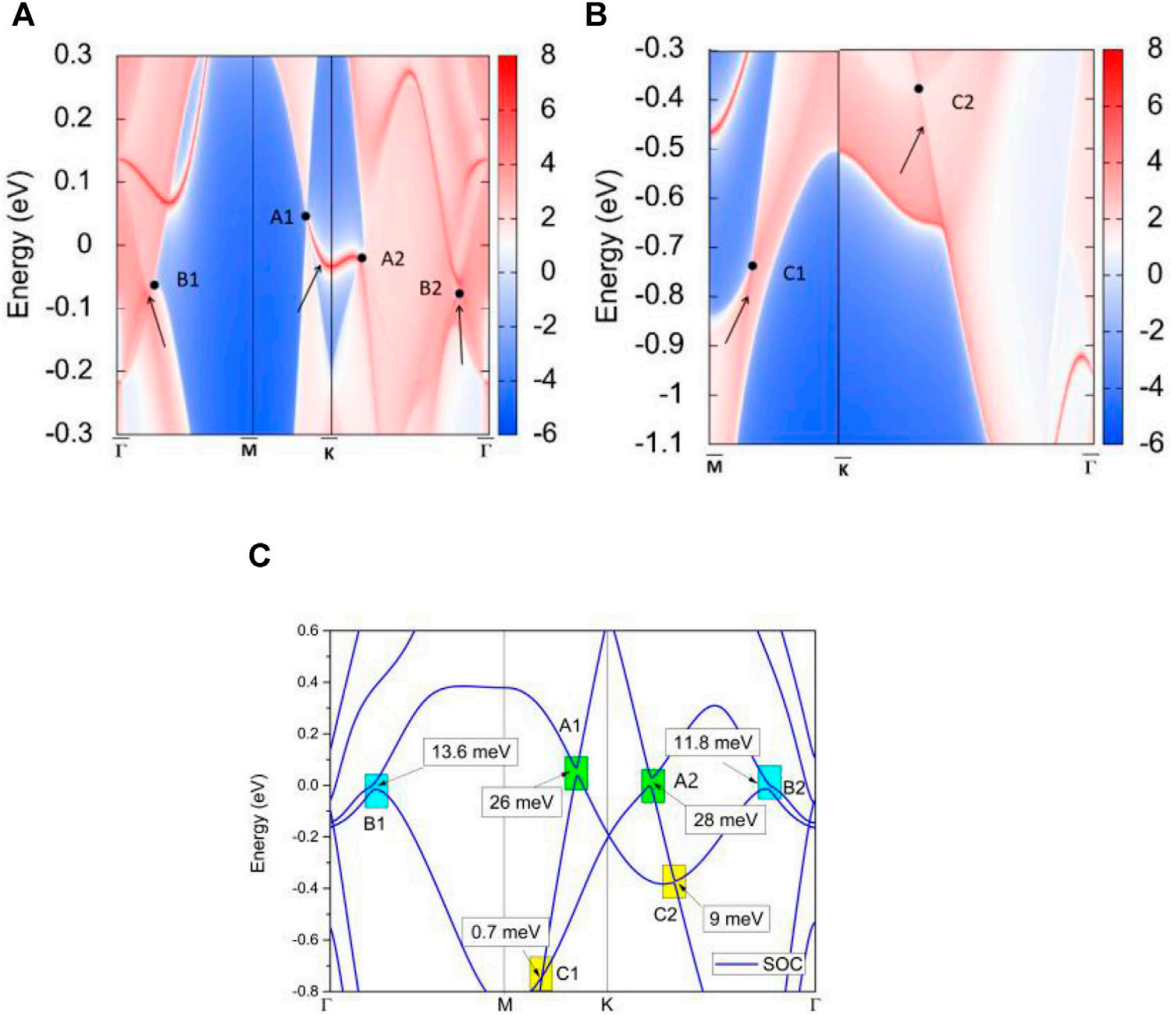
**(A,B)** The projected spectrum on the (001) surface of TiTe. The BCPs—A1, A2, B1, B2, C1, and C2—and the drum-head-like surface states are highlighted by the circles and arrows, respectively. **(C)** The band structure of TiTe under spin-orbit coupling.

In this section, we provide strong evidence for the appearance of the NLs in the three regions. NL materials usually host drum-head-like (D-H-L) surface states (Wang et al., 2020d) connected to the ban-crossing points, one of its most important characters. Figures 8A,B show the projected spectrum on the TiTe (001) surface. We use the black circles to indicate the positions of the BCPs. The D-H-L surface states, connected to the BCPs and marked by arrows, can be observed. Such obvious D-H-L surface states in TiTe benefit the experimental detection. It is expected that angle-resolved optical emission spectroscopy (ARPES) can be used to detect the D-H-L surface states in TiTe directly.

## Effect of SOC

The SOC usually induces a gap in the BCPs in most NL materials. The SOC-induced gap is particularly very large (50–200 meV) when the material contains heavy elements (Huang et al., 2016; Yamakage et al., 2016; Wang et al., 2020e), which significantly damages the intrinsic electronic properties of the NLs. Figure 8C shows the band structure with SOC. Therefore, we conclude that the SOC-induced gap for these band-crossings is smaller than 28 meV and within the acceptable limits, reflecting that TiTe is an ideal NL material with robust resistance to the effects of SOC.

## Conclusion

We prove the existence of I, II, and hybrid types of NLs in TiTe at the ground state. Moreover, TiTe is shown to be a dynamic and mechanically stable material using first-principle calculations. Furthermore, it is proved to be an ideal NL material with two type I NLs: one Γ-centered type II NL and two hybrid-type NLs in the k_z_ = 0 plane. The BCPs are robust to the SOC, and the SOC-induced gaps are quite small. The D-H-L surface states can be observed in (001) surface of the TiTe. We expect that the NLs and the (001) surface states of TiTe can be verified in an experiment.

## Data Availability

The original contributions presented in the study are included in the article/Supplementary Material, further inquiries can be directed to the corresponding authors.

## Footnotes

1
https://materialsproject.org/materials/mp-567832
