# Supplementary material for: Various Nodal Lines in P63/mmc-type TiTe Topological Metal and its (001) Surface State
Source: Front Chem. 2021 Sep 28;9:755350. doi: 10.3389/fchem.2021.755350 (PMC8510513; doi:10.3389/fchem.2021.755350)
Supplement: Supplementary file 1 [file DataSheet1.doc]

Methods

The first-principles method was selected in this work to calculate the electronic structure and the topological properties of the TiTe material, as implemented in the Vienna Ab initio Simulation Package [S1]. We adopted the generalized gradient approximation (GGA) [S2] of the Perdew–Burke–Ernzerhof (PBE) [S3] functional as the exchange-correlation potential. Moreover, we set the cutoff energy as 600 eV, and sampled the Brillouin zone (BZ) via a Monkhorst–Pack k-mesh with a size of 10 × 10 × 4. We set the energy/force convergence criteria as 10^-6^ eV/10^-3^ eV. On the basis of the iterative Green’s function method, as implemented in the Wannier Tools package [S4], the surface states of the TiTe (001) plane were simulated. The lattice dynamic calculations are performed to obtain the phonon dispersion of TiTe at equilibrium lattice constants in the PHONOPY package [S5] using density functional perturbation theory.

References

[S1] Hafner, J. (2008). Ab-initio simulations of materials using vasp: Density-functional theory and beyond, Journal of Computational Chemistry, 29, 2044.

[S2] Perdew, J. P., Burke, K., & Ernzerhof, M. (1996). Generalized gradient approximation made simple. Physical Review Letters, 77(18), 3865.

[S3] Perdew, J. P., Burke, K., & Ernzerhof, M. (1998). Perdew, burke, and ernzerhof reply. Physical Review Letters, 80(4), 891.

[S4] Wu, Q., Zhang, S., Song, H. F., Troyer, M., & Soluyanov, A. A. (2018). WannierTools: An open-source software package for novel topological materials. Computer Physics Communications, 224, 405-416.

[S5] Togo, A., & Tanaka, I. (2015). First principles phonon calculations in materials science. Scripta Materialia, 108, 1-5.
